# The gestural repertoire of the wild bonobo (*Pan paniscus*): a mutually understood communication system

**DOI:** 10.1007/s10071-016-1035-9

**Published:** 2016-09-15

**Authors:** Kirsty E. Graham, Takeshi Furuichi, Richard W. Byrne

**Affiliations:** 1 School of Psychology and Neuroscience, University of St Andrews, St Andrews, UK; 2 Primate Research Institute, University of Kyoto, Kyoto, Japan

**Keywords:** Gesture, Understood repertoire, Expressed repertoire, Bonobo, Chimpanzee

## Abstract

In animal communication, signallers and recipients are typically different: each signal is given by one subset of individuals (members of the same age, sex, or social rank) and directed towards another. However, there is scope for signaller–recipient interchangeability in systems where most signals are potentially relevant to all age–sex groups, such as great ape gestural communication. In this study of wild bonobos (*Pan paniscus*), we aimed to discover whether their gestural communication is indeed a mutually understood communicative repertoire, in which all individuals can act as both signallers and recipients. While past studies have only examined the *expressed* repertoire, the set of gesture types that a signaller deploys, we also examined the *understood* repertoire, the set of gestures to which a recipient reacts in a way that satisfies the signaller. We found that most of the gestural repertoire was both expressed and understood by all age and sex groups, with few exceptions, suggesting that during their lifetimes all individuals may use and understand all gesture types. Indeed, as the number of overall gesture instances increased, so did the proportion of individuals estimated to both express and understand a gesture type. We compared the community repertoire of bonobos to that of chimpanzees, finding an 88 % overlap. Observed differences are consistent with sampling effects generated by the species’ different social systems, and it is thus possible that the repertoire of gesture types available to *Pan* is determined biologically.

**Electronic supplementary material:**

The online version of this article (doi:10.1007/s10071-016-1035-9) contains supplementary material, which is available to authorized users.

## Introduction

Animal communication includes a vast array of signalling systems, ranging from the warning colouration of noxious insects to the complexity of human language. Language is exceptional in many ways, not least for being a system of largely arbitrary signals that an entire population has the capacity to use and understand. In many other communication systems, the signals that an individual can use are strictly limited by their age, sex, or social position. Thus, the visual displays of lekking bird species (Endler and Thery 1996), peacock spiders (Girard et al. 2011), smooth newts (Halliday 1974), and ring-tailed lemurs (Sauther et al. 1999) are produced only by adult males and directed towards females. In other species, females direct visual signals towards males, for example, the bioluminescent signals of fireflies (Lewis and Cratsley 2008) or cowbird wing strokes (West and King 1988). For cowbirds, this visual signal given only by females is in response to a vocal signal given only by males (West and King 1988), illustrating that although both sexes are signallers and recipients, they are not signallers and recipients of the same signal.

Great ape gestural communication might be more similar to language, in the sense that no such restrictions have been noted; signallers and recipients are in principle interchangeable for all signals, because gestures are movements of limbs, head, or body, which could potentially be produced by any one individual. Limitations on interchangeability may nevertheless exist, as in other species. Obvious physical requirements may shape usage: for example, only adult females carry infants and juveniles and therefore may be the only ones to deploy gestures that signal “climb on my back”. Less trivially, gestures may be limited to subsets of individuals because of subtle differences in adapted traits or developmental experience. A major aim of the current research is to determine whether great ape gestural communication genuinely shows interchangeability.

To date, great ape gestural research has focused exclusively on the *expressed* repertoire—the set of gesture types that an individual deploys. Expressed repertoires have been described for all great ape species in captivity (Call and Tomasello 2007; Genty et al. 2009; Cartmill and Byrne 2010) and for wild chimpanzees (Hobaiter and Byrne 2011). Here we also examine the *understood* repertoire—the set of gesture types that an individual receives and subsequently understands. Great apes intentionally deploy gestures (Tomasello et al. 1989; Leavens et al. 2005); in first-order intentional communication, the signaller uses gestures in order to change the behaviour of the recipient (Dennett 1983), and the meaning of a gesture can be determined by its apparently satisfactory outcome (ASO), which is the reaction of the recipient that satisfies the signaller, confirming that the signaller’s intended goal was met (e.g. Cartmill and Byrne 2010; Hobaiter and Byrne 2014). To determine whether a gesture is part of an ape’s understood repertoire, we take the converse approach: if a recipient reacts to a gesture with an ASO, then it can be taken to have understood that gesture.

Studying gestural communication in the wild enables a better estimate of the repertoire than in captivity, because the range of circumstances in which communication occurs is not artificially constrained (e.g. by food provisioning, veterinary interventions, restrictions of group composition, contraception). In an 18-month study on wild chimpanzees, the community repertoire was shown to be close to asymptote at 66 gesture types, a much greater estimate than in previous captive studies (Hobaiter and Byrne 2011). Nevertheless, the average individual (expressed) repertoire size was still only 10 gesture types, and individual repertoires were shown to be far from asymptote. We therefore propose that it is appropriate to maximize the available evidence by reporting both the understood and expressed repertoires of great apes, in order to more accurately chart individual repertoires and to detect possible differences of usage among species. Comparing expressed and understood gestural usage should allow us to detect limitations on signaller/recipient interchangeability.

This paper is the first to catalogue the community repertoire for a wild community of bonobos, and so it also gives the first opportunity to compare with a community repertoire for wild chimpanzees, in order to investigate the degree to which they overlap. The chimpanzee repertoire appears to be largely species typical, biologically driven rather than acquired on an individual basis (Hobaiter and Byrne 2011; note that some gesture types may be learned socially: Halina et al. 2013), and we examine whether the same is true of the bonobo. Comparison with the other *Pan* species allows the possibility of a shared *Pan* repertoire to be explored, and by studying both of our closest living relatives, we may be better able to understand the evolution of human language.

## Methods

### Study sites and subjects

Fieldwork was conducted at Wamba, Luo Scientific Reserve, Democratic Republic of Congo (00°10′N, 22°30′E). We followed two neighbouring communities of wild bonobos: E1 group (*n* = 39) has been habituated since 1974, and *P* group (*n* = 30) has been habituated since 2010. In 2014, the total sample size was 63 individuals, with 28 adults (16 females, 12 males), 12 adolescents (7 females, 5 males), 9 juveniles (6 females, 3 males), and 14 infants (8 females, 6 males). In 2015, the total sample size was 64 individuals, with 30 adults (18 females, 12 males), 8 adolescents (3 females, 5 males), 10 juveniles (7 females, 3 males), and 16 infants (10 females, 6 males).

### Data collection

This study was approved by the School of Psychology and Neuroscience Ethics Committee at the University of St Andrews, and permission to conduct the study was granted by the Ministère de la Recherche Scientifique et Technologie in the Democratic Republic of the Congo. Data collection took place from February to June 2014 and January to June 2015. We conducted daily observations from approximately 05:50 to 12:00, with a rough schedule of 4-day working and 1-day off, observing bonobos on a total of 204 days, amounting to ~1159 h of observation time.

We used focal behaviour sampling to film social interactions. Filming began whenever two or more individuals came within 5 m range of each other, in order to catch the beginning of social interactions. We recorded video footage using a Panasonic HDC-SD90 video camera, which has a pre-record feature that continually records the previous 3 s. Each day after returning from daily follows, we imported footage and sorted it into a clip directory using FileMaker Pro.

### Video coding

Gestures were defined as discrete, mechanically ineffective physical movements of the body observed during periods of intentional communication, including movements of the whole body, limbs and head, but not facial expressions or static body postures. We created a separate coding sheet in Filemaker Pro for each gesture instance, recording the following information: signaller, recipient, signaller age/sex, recipient age/sex, gesture type, part of sequence, part of bout, audience checking, response waiting, persistence, and signaller apparently satisfied. *Signaller* is the gesturing individual, and *recipient* is the individual to whom the gesture is directed. Age groups are taken from Hashimoto’s bonobo age classification (Hashimoto 1997): infant (<4 years), juvenile (4–7 years), adolescent (8–14 years), and adult (15+ years). *Gesture type* is defined by the physical form of the gesture, where possible following definitions are used with the chimpanzee (Hobaiter and Byrne 2011), but adding new definitions for gesture types that have not been reported in the chimpanzee. A *sequence* is defined as a series of gesture instances given by one individual, separated by <1 s. A *bout* is defined as a series of gesture instances or sequences given by one individual, separated by pauses of >1 s. *Audience checking* is when the signaller turns to face the recipient before or during gesturing. *Response waiting* is when the signaller pauses for >1 s after gesturing while maintaining visual contact. *Persistence* is when the signaller continues to gesture at the same recipient. Each instance of a gesture was required to meet at least one criterion for intentionality before it was accepted for analysis: audience checking, response waiting, or persistence.

For the expressed repertoire, we included all gesture types that an individual deployed. The understood repertoire, however, was not simply the gesture types that an individual received, but the gesture types that they understood. We took it that the recipient understood a gesture instance if the recipient reacted with an apparently satisfactory outcome (ASO)—i.e. a reaction that satisfied the signaller, as shown by cessation of gesturing. The signaller should start to react during gesturing or immediately following cessation of gesturing. Note that an ASO must be a *change* in behaviour: if the recipient remains in the same state and the signaller stops gesturing, there was no change in behaviour from the recipient, and thus, we coded “No response”, not “ASO”. For gestures occurring in sequences, if the recipient responded to the sequence with an ASO, that ASO was assigned to all gestures in the sequence, not only the final gesture instance in the sequence.

### Inter-observer reliability

To corroborate the accuracy of our video coding, a second experienced coder, Dr Catherine Hobaiter, coded 100 gesture instances for the following information: gesture type, persistence, and signaller apparently satisfied. We calculated inter-observer reliability using Cohen’s Kappa, revealing agreement for all variables (gesture type *K* = 0.87, persistence *K* = 0.70, and signaller apparently satisfied *K* = 0.63).

## Results

### Bonobo and chimpanzee community repertoires

**Fig. 1:**
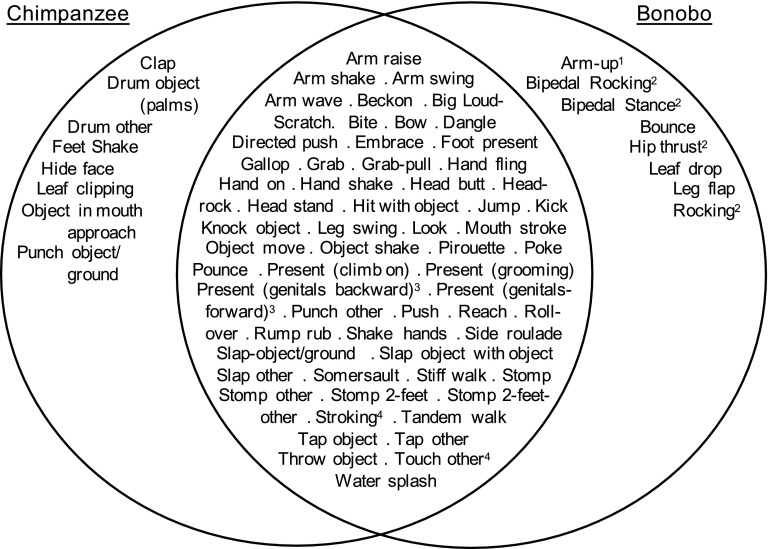
*Venn diagram* showing the gesture types used by chimpanzees (Hobaiter and Byrne 2011) and bonobos. Eighty-eight per cent of the gestures overlap. (1) Seen in chimpanzees at Bossou, not reported at Budongo (Catherine Hobaiter, personal communication). (2) Seen in chimpanzees at Budongo, subsequent to Hobaiter and Byrne 2011 (Catherine Hobaiter, personal communication). (3) We split *Present (genitals backward)* and *Present (genitals forward)*, which were combined as *Present (sexual)* in Hobaiter and Byrne 2011. (4) We split *Stroking* and *Touch other*, which were combined as *Present (sexual)* in Hobaiter and Byrne 2011

We recorded 4256 intentionally produced gesture instances used within E1 and P groups, which we classified into 68 gesture types (Online Resource 1): the bonobo community repertoire. For wild chimpanzees, 66 gesture types have been reported (Hobaiter and Byrne 2011), but in the present analysis, we split two of the categories used in that study, *Touch other* to *Touch other* and *Stroking,* and *Present (sexual)* to *Present (genitals forward)* and *Present (genitals backward)*, so the comparable chimpanzee repertoire is 68 gesture types. With this correction, 60/68 bonobo gesture types were shared with chimpanzees, an 88 % overlap (Fig. 1). As noted in the caption to Fig. 1, several of the “bonobo-specific” gesture types have been discovered in chimpanzees subsequent to Hobaiter and Byrne’s publication.

### Expressed and understood repertoires

The mean expressed repertoire for individual bonobos was 14.40 ± SD 7.69 gesture types, *N* = 65 (range 1–35); the mean understood repertoire was 10.48 ± SD 5.86 gesture types, *N* = 65 (range 0–30). Combining these estimates gave a mean overall repertoire of 18.82 ± SD 9.07 gesture types, *N* = 65 (range 1–42). A one-way paired *T* test shows that the overall repertoire is significantly larger than the expressed repertoire (*t*64 = −11.29, *p* < 0.01).

**Fig. 2:**
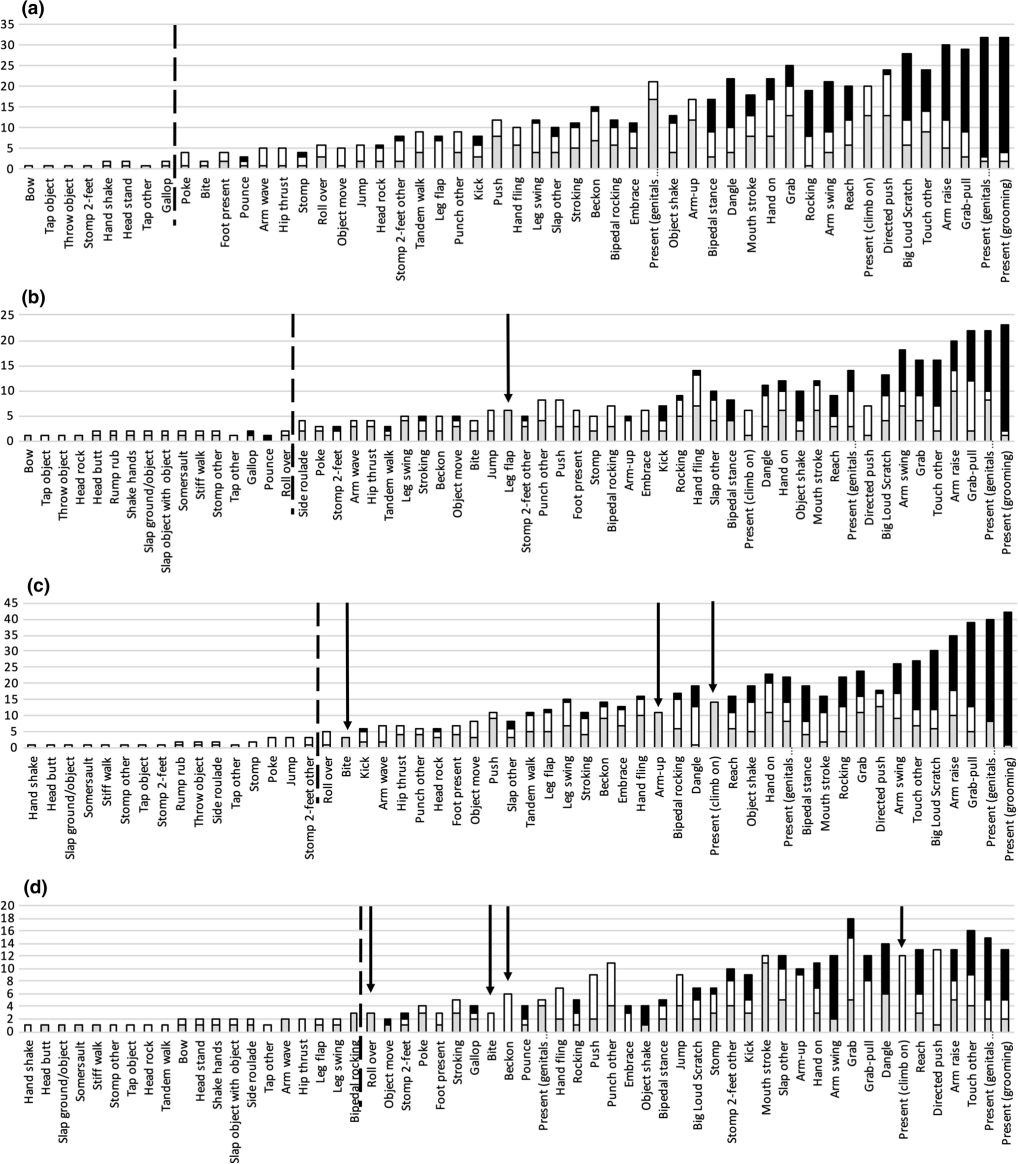
Stacked histograms with number of individuals on the* y-axis*, showing for each gesture type the number of individuals who express a gesture (*in grey*), understand a gesture (*in white*), or both express and understand a gesture (*in black*). Histograms are divided by sex: **a** female, **b** male; and by age: **c** adult and adolescent, **d** Juvenile and infant. Gesture types are arranged on the* x-axis* from *left* to *right* in increasing number of gesture instances. Gesture types to the *left* of the *black dashed line* have <3 gesture instances; those to the *right* have >3 gesture instances. The *black arrows* point out gesture types that are exclusively either expressed or understood

In order to examine whether any gesture types were primarily produced by one subset of individuals but understood by another, we matched the gesture instances in individuals’ expressed and understood repertoires. For this analysis, we restricted the data to gesture instances (including those in sequences) that were understood, giving 2694 gesture instances and 60 gesture types. First, we grouped individuals by sex (female and male) and plotted the number of individuals that express, understand, or both express and understand each gesture type (Fig. 2a, b; Online Resource 2). Analysis was restricted to gesture types that were observed more than three times, giving 47 gesture types. All were both expressed and understood by members of both sexes, with the exception of *Leg flap*, which was expressed by but not given to males.

We then grouped individuals into age groups (adult + adolescent and juvenile + infant) and plotted the number of individuals that express, understand, or both express and understand each gesture type (Fig. 2c, d). Again analysis was restricted to the 47 gesture types observed more than three times. Most gesture types were both expressed and understood by members of the two age groups. However, three gesture types, *Bite*, *Arm up*, and *Present (climb on)*, were expressed by but not given to adults and adolescents. Three gesture types, *Bite*, *Beckon*, and *Present (climb on)*, were received and understood, but not expressed by juveniles and infants; one gesture type, *Roll over*, was expressed by but not given to juveniles and infants.

Finally, we calculated an index for each gesture type:

$$= \frac{{\#\, {\text{individuals}} \;{\text{that}}\; {\text{both}}\;{\text{expressed }}\;{\text{and}}\;{\text{understood}}\;{\text{gesture}}}}{{\# \,{\text{individuals}}\;{\text{that}}\;{\text{either}}\;{\text{expressed}}\;{\text{or}}\;{\text{understood}}\;{\text{gesture}} }}$$

**Fig. 3:**
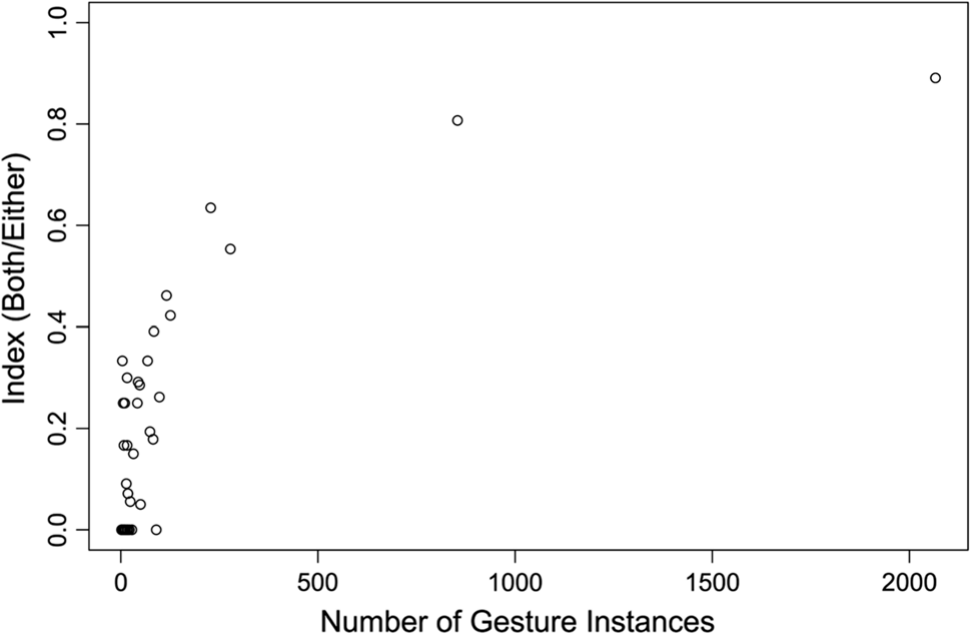
Index (number of individuals that both express and understand number of individuals that either express or understand) expressed against the number of gesture instances for each gesture type

Provided sufficient data are available, values closer to 1 would show that most individuals both use and understand the gesture type, whereas values closer to 0 reveal gesture types that are typically used and understood by different individuals. Index values ranged from 0.00 to 0.89 (Online Resource 2). We plotted the index (dependent variable) against the total number of gesture instances (independent variable) for each gesture type (Fig. 3). As the number of gesture instances increases, so does the index, suggesting that for most gestures the index is a serious underestimate of signaller/recipient interchangeability. However, Fig. 3 suggests that when 2000 instances have been sampled, an asymptote of around 90 % overlap between signallers and recipients should be expected, i.e. 90 % of the community will both use and understand every gesture.

## Discussion

When linguists and developmental psychologists study an individual’s vocabulary, they not only look at the words an individual uses (the productive vocabulary), but also the words that an individual understands (the receptive vocabulary). And yet, past studies on primate gestural communication have focused exclusively on the former, the so-called expressed repertoire. In order to examine the role of an individual as a signaller *and* a recipient, we also need to look at the gestures that they receive and understand. Here, we used responding with an ASO as a measure of understanding a gesture and were therefore able to chart the understood repertoires of individual bonobos.

Comparison of females and males revealed that members of both sexes expressed and understood all gesture types observed three or more times, with the exception of one gesture that was never directed at a male. Age groups showed slightly more exclusive gesture types: three that were expressed by but not directed at adults or adolescents; three that were received and understood but not expressed by juveniles or infants; and one that was expressed by but not directed at juveniles or infants. That is, out of 47 gesture types that were expressed and understood more than three times, 42 of them were both expressed and understood by all age groups. A possible explanation for the few apparently exclusive gesture types may be paucity of data, and differences may disappear with more gesture instances; consistent with that hypothesis, relatively few instances were recorded in total for *Roll over* (10)*, Bite* (12), or *Beckon* (26), whereas it seems a less likely explanation for *Arm up* (50 instances) and *Present (climb on)* (90). Alternatively, differences in signallers and recipients between age groups may reflect the different requirements during those life stages: an individual’s active repertoire may change through ontogeny, eventually including all gestures in the community repertoire. The interchangeability of signaller and recipient between sexes supports this conjecture: over their lifetime, males and females will both have opportunities to use each gesture type.

When examining whether individuals expressed, understood, or both expressed and understood each gesture type, we found that as the number of gesture instances increased, so did the ratio of individuals that both expressed and understood a gesture type. Our graph appeared to reach asymptote at 90 % with >2000 gesture instances per gesture type. That this estimate may be a reasonable one is illustrated by the case of gesture type *Present (Climb on)*. The gesture *Present (Climb on)* is how a mother bonobo gets an infant to cling on to her body for travel, yet even this gesture type, which seems specifically useful for mothers and their offspring, proved not to be used by adult females alone. One adult male carried a juvenile male consistently for ~1 month (intermittently for ~3 months), during which time he employed *Present (climb on)* to encourage the juvenile to cling on to his body for travel. It would appear that absence from the expressed repertoire may normally represent limited opportunity to use a gesture type, rather than absence from the actual repertoire.

All this evidence indicates a mutually understood communication system that is, unlike many other visual displays, largely unconstrained by sex or age, and wherein all individuals are potentially signallers and recipients for all gestures. While a small minority of gesture types might be learned socially or by ritualization (Halina et al. 2013), the general interchangeability of signaller and recipient is difficult to reconcile with the one-way gestures predicted by “ontogenetic ritualization”. Mutual understanding is a vital feature of human language that all shared-language users know the same signals and meanings of the signals. Here we have shown that, like in language, all individuals are able to use and understand the same signals. Future research should examine whether these signals mean the same thing for all individuals.

Bonobo gestural communication is therefore an intentional, flexible, mutually understood communicative system: a conclusion that is made more striking by the fact that 88 % of their repertoire overlaps with that of the chimpanzee. Actually, the bonobo–chimpanzee gestural overlap may be even greater. Several gesture types not reported by Hobaiter and Byrne (*Bipedal rocking, Bipedal stance, Hip thrust, Rocking, Swat*) have since been seen in chimpanzees at Budongo, Uganda, and one gesture type (*Arm up*) has been seen at Bossou, Guinea (Catherine Hobaiter, personal communication). Including these gesture types raises our total to 64 gesture types shared with chimpanzees—a 96 % overlap. That leaves 3 gesture types (*Bounce, Leaf drop, Leg flap*) as apparently bonobo-exclusive gesture types. All three of these gesture types are used in a sexual context. Bonobos and chimpanzees have markedly different social behaviour, which might plausibly be reflected in their gestural communication, with a greater repertoire of socio-sexual signals. Bonobo females engage in female–female sexual behaviour, genito-genital rubbing (Idani 1991; Hohmann and Fruth 2000) and are also more central to the group (Furuichi 2011). This means that bonobos, in particular female bonobos, may have more opportunity to use sexual solicitation gestures, raising another possibility: that the differences between the bonobo and chimpanzee gestural repertoires may simply be an artefact of lack of data rather than the complete absence of a gesture type. Likewise, several of the chimpanzee gesture types that are absent in the bonobo repertoire are related to male displays and dominance, behaviour that is far less prevalent in the bonobo.

The bonobo and chimpanzee repertoire therefore seem, to a very considerable extent, to be *Pan*-typical. However, the question remains: Do bonobo and chimpanzee gestures mean the same thing? Despite the differences in social behaviour between bonobos and chimpanzees, differences in the gestural repertoire are minor and perhaps artefactual: but while the gesture form might be biologically fixed, the meaning may not be and remains a potential source of inter-species differences. Future studies will need to compare gesture meanings between bonobos and chimpanzees, in order to discover how profoundly biological their repertoire is. Comparison of our two closest living relatives is also important for understanding the evolution of language, with many of the component features of language, e.g. mutual understanding and intentionality, being present in their gestural communication.

## Electronic supplementary material

Below is the link to the electronic supplementary material.

Supplementary material 1 (DOCX 23 kb)

Supplementary material 2 (DOCX 1546 kb)
