# Supplementary material for: The gestural repertoire of the wild bonobo (Pan paniscus): a mutually understood communication system
Source: Anim Cogn. 2016 Sep 15;20(2):171–7. doi: 10.1007/s10071-016-1035-9 (PMC5306194; doi:10.1007/s10071-016-1035-9)
Supplement: Supplementary file 1 — Supplementary material 1 (DOCX 23 kb) [file 10071_2016_1035_MOESM1_ESM.docx]

The gestural repertoire of the wild bonobo (*Pan paniscus*): Expressed and understood repertoires, Animal Cognition, Kirsty E Graham and Richard W Byrne; School of Psychology & Neuroscience, University of St Andrews, UK, rwb@st-andrews.ac.uk

| **Bonobo gesture list** | **Hobaiter & Byrne 2011** | **Description of gesture types** |
| --- | --- | --- |
| Arm raise | Arm raise | “Raise arm(s) and/or hand(s) vertically in the air” |
| Arm shake^1^ | Arm shake | “Small repeated back and forth motion of the arm” |
| Arm swing | Arm swing | “Large back and forth movement of the arm(s) held below the shoulder” |
| Arm up^2^ | - | **NEW: Extend straight arm(s) out to side and away from body** |
| Arm wave | Arm wave | “Large repeated back and forth movement of the arm(s) raised above the shoulder” |
| Beckon | Beckon | “Hand is moved in an upwards sweep from the elbow or wrist towards the signaller” |
| Big loud scratch | Big loud scratch | “Loud exaggerated scratching movement on the signaller’s own body” |
| Bipedal rocking^3^ | - | **NEW: Stand or walk bipedal, rock forward and back or side to side, repeated (includes rare ‘Quadrupedal rocking’)** |
| Bipedal stance^3^ | - | **NEW: Stand bipedal, arms out to side, back arched** |
| Bite | Bite | “Recipient’s body is held between the teeth of the signaller” |
| Bounce^2^ | - | **NEW: Standing quadrupedal, bend elbows and knees to move up and down repeatedly** |
| Bow^1^ | Bow | “Signaller bends forward from the waist while standing [bipedal]” |
| - | Clap | “Both palms move towards each other and are brought together with an audible contact” |
| Dangle | Dangle | “To hang from one or both arms from a branch above another individual; this is audible as there is normally significant disturbance of the canopy” |
| Directed push | Directed push | “A light short non-effective push that indicates a direction of desired movement, immediately followed by the recipient moving as indicated” |
| - | Drum object (palms) | “Short, hard, audible contact of alternate palms against an object” |
| - | Drum other | “As ‘Drum object (palms)’ but contact is with recipient’s body” |
| Embrace | Embrace | “Signaller wraps [one or] both arms around the recipient and maintains physical contact” |
| - | Feet shake | “Repeated back and forth movement of feet from the ankles” |
| Foot present | Foot present | “Sole of the foot is presented to the recipient” |
| Gallop | Gallop | “An exaggerated running movement where the contact of the hands and feet is deliberately audible” |
| Grab | Grab | “The hand[(s)] is[/are] firmly closed over part of the recipient’s body” |
| Grab-pull | Grab-pull | “As ‘Grab’ but closed hand contact is maintained and a force exerted to move the recipient from their current position” |
| Hand fling | Hand fling | “Rapid movement of the hand or arm in the direction of the recipient”  **Includes “Swat” - NEW: Swipe hand in sharp downward motion without making contact with the recipient** |
| Hand on | Hand on | “Palm[(s)] of the hand[(s)] is[/are] placed on the recipient, contact lasts for more than 2 s” |
| Hand shake^1^ | Hand shake | “Repeated back and forth movement of hand from the wrist” |
| Head butt | Head butt | “Head is briefly and firmly pushed into the body of the recipient” |
| Head rock | Head nod | “Repeated back and forth [or side to side] movement of the head” (includes ‘Head shake’)^4^ |
| Head stand | Head stand | “Signaller bends forward and places head on the ground” |
| - | Hide face | “Face is hidden by the hands and/or arms” |
| Hip thrust^3^ | - | **NEW: Sitting, crouching, or standing, thrust hips forward (single or repeated)** |
| Hit with object^1^ | Hit with object | “An object is brought into short, hard contact with the body of the recipient” |
| Jump | Jump | “While bipedal, both feet leave the ground simultaneously, accompanied by horizontal displacement through the air” |
| Kick | Kick | “Foot is brought into short hard contact with the recipient’s body in a movement from the hip with a horizontal element (for vertical see ‘Stomp other’)” |
| Knock object^1^ | Knock object | “Back of the hand or knuckles are brought into short, hard, audible contact with an object” |
| - | Leaf clipping | “Strips are torn from a leaf (or leaves) held in the hand using the teeth; produces a conspicuous sound” |
| Leaf drop | - | **NEW: Pick leaf(s) and drop it, usually signaller is above recipient** |
| Leg flap | - | **NEW: Sitting with knees bent, open and close one or both legs to side (single or repeated)** |
| Leg swing | Leg swing | “Large back and forth movement of the leg from the hip” |
| (Look^5^) | Look | “Signaller holds an eye-contact position with the recipient–minimum duration 2 s” |
| Mouth stroke | Mouth stroke | “Signaller’s palm and fingers are repeatedly run over the mouth area of the recipient” |
| - | Object in mouth approach | “Signaller approaches recipient while carrying an object in the mouth (e.g. a small branch)” |
| Object move | Object move | “Object is displaced in one direction, contact is maintained through movement” (includes ‘Branch drag’)^4^ |
|  |  |  |
| Object shake | Object shake | “Repeated back and forth movement of an object” (includes ‘Object shake tandem’)^4^  **Object shake tandem: As ‘Object shake’ but object is in contact with the recipient** |
|  |  |  |
| Pirouette^1^ | Pirouette | “Signaller turns around their body’s vertical axis while also displacing along the ground” |
| Poke | Poke | “Firm, brief push of one or more fingers into the recipient’s body” |
| Pounce | Pounce | “Signaller displaces through the air to land quadrupedally on the body of the recipient” |
| Present (climb on)^6^ | Present climb on me | “Arm or leg is extended to young recipient in order to facilitate them climbing onto the signaller’s body (normally mother to infant)” |
| Present (grooming)^6^ | Present grooming | “Body is moved to deliberately expose an area to the recipient’s attention which is immediately followed by grooming of the area” |
| Present (genitals backward) | Present sexual | “Signaller approaches recipient backwards, exposing the swelling or anus to the recipient’s face”  **Present genitals forwards: the signaller sits and spreads their limbs displaying their genital swelling or erect penis** |
| Present (genitals forward) |  |  |
| - | Punch object/ground | “Movement of whole arm, with short, hard, audible contact of the closed fist to an object or the ground” |
| Punch other | Punch other | “As ‘Punch object/ground’ but contact is with recipient’s body” |
| Push | Push | “Palm in contact with recipient’s body and force is exerted in an attempt to displace recipient” |
| Reach | Reach | “Arm extended to the recipient with hand in an open, palm upwards[, downwards, or sideways] position” (modified from Hobaiter & Byrne 2011 to include ‘Reach – palm down’, ‘Reach – palm side’, ‘Reach – palm up’, and ‘Reach – wrist first’)^4^ |
| Rocking^3^ | - | **NEW: Sitting, rock forward and back or side to side, repeated** |
| Roll over | Roll over | “The signaller rolls onto their back exposing their stomach, normally accompanied by repeated movements of the arms and/or legs” |
| Rump rub^1^ | Rump rub | “Push/rub rump against the body/swelling of recipient” |
| Shake hands | Shake hands | “Signaller grasps recipient’s hand in their own hand and then makes small repeated back and forth movements from the wrist” |
| Side roulade | Side roulade | “Body is rotated around the head-feet axis while lying on the ground with horizontal displacement along the ground” |
| Slap object/ground^1^ | Slap object | “Movement of the arm from the shoulder with hard, short contact of the palm of the hand to an object [or the ground]” |
| Slap object/ground with object^1^ | Slap object with object | “As ‘Slap object’ but the hand holds an object which is brought into contact with another object [or the ground] (e.g. a branch is slapped against a tree) |
| Slap other | Slap other | “As ‘Slap object’ but the palm is brought into contact with the recipient’s body” |
| Somersault | Somersault | “Signaller’s body is curled into a compact position on the ground, and rolled forwards so the feet are brought over the head and returned to a sitting position” |
| Stiff walk^1^ | Walk (stiff) | “Walk quadrupedally with a slow, exaggerated movement” |
| Stomp | Stomp | “Sole of the foot is lifted vertically and brought into a short, hard, audible contact with the surface being stood upon (e.g. ground or a branch)” |
| Stomp other | Stomp other | “As ‘Stomp’ but contact is made with recipient |
| Stomp 2-feet | Stomp 2-feet | “As ‘Stomp’ but both feet used, normally alternately” |
| Stomp 2-feet other | Stomp 2-feet other | “As ‘Stomp 2-feet’ but contact is made with the recipient” |
| Stroking | - | **NEW: Run palm of hand gently over recipient’s body repeatedly**  (in Hobaiter & Byrne 2011, ‘Stroking’ was included in ‘Touch other’) |
| Tandem walk | Tandem walk | “[Signaller] positions arm over the body of the recipient and both walk forward while maintaining position” |
| Tap object^1^ | Tap object | “Movement of the arm from the wrist of elbow, with firm, short contact of the fingers to the object (single/multiple)” |
| Tap other | Tap other | “As Tap object’ but contact is with the recipient’s body” |
| Throw object^1^ | Throw object | “Object is moved and released so that there is displacement through the air after moment of release” |
| Touch other | Touch other | “Light contact with the palm and/or fingers on the body of the recipient, contact under 2 s” (as for Hobaiter & Byrne 2011, ‘Touch other’ includes ‘Knock other’)^4^  **Knock other: As “Knock object” but make contact with the recipient** |
| Water splash^1^ | Water splash | “Hand is moved vigorously through the water so that there is audible displacement of the water” |
| **= 68 gesture types** | **= 66 gesture types** |  |

*Online Resource 1.*

Table showing the bonobo gestural repertoire compared to the chimpanzee gestural repertoire described by Hobaiter and Byrne, 2011. Clarifications to the descriptions in Hobaiter and Byrne, 2011 have been marked in square brackets: [ ]. New gesture types and gesture types that were not described in Hobaiter and Byrne, 2011 are marked in **bold text**.

^1^ Observed at Wamba fewer than 2 times.

^2^ Seen in chimpanzees at Bossou, not reported at Budongo (Catherine Hobaiter, personal communication).

^3^ These gesture types have now been seen at Budongo, but were not reported in Hobaiter & Byrne, 2011 (Catherine Hobaiter, personal communication).

^4^ These gesture types were lumped for comparison with Hobaiter & Byrne, 2011. Future studies should use ASOs to determine whether they have different meanings.

^5^ ‘Look’ behaviour was observed in bonobos at Wamba, but not considered intentional.

^6^ ‘Present climb on me’ and ‘Present grooming’ are used for comparison with the chimpanzee. We propose using ‘Present body part’, in future, to disassociate the gesture form from any presumptive meaning.
