# Supplementary material for: The gestural repertoire of the wild bonobo (Pan paniscus): a mutually understood communication system
Source: Anim Cogn. 2016 Sep 15;20(2):171–7. doi: 10.1007/s10071-016-1035-9 (PMC5306194; doi:10.1007/s10071-016-1035-9)
Supplement: Supplementary file 2 — Supplementary material 2 (DOCX 1546 kb) [file 10071_2016_1035_MOESM2_ESM.docx]

The gestural repertoire of the wild bonobo (*Pan paniscus*): Expressed and understood repertoires, Animal Cognition, Kirsty E Graham and Richard W Byrne; School of Psychology & Neuroscience, University of St Andrews, UK, rwb@st-andrews.ac.uk

*Online Resource 2*.

Table showing the number of gesture instances per gesture type that each individual either expresses, or understands, or both expresses and understands. Gesture types that an individual (sorted by sex-age groups) only expresses are in yellow; only understands are in blue; or both expresses and understands are in green. At the bottom, shows the overall repertoire size for each individual. On the right side, shows (a) number of individuals that both express and understood the gesture type, (b) the number of individuals that either express or understand the gesture type, (c) the index of a/b, and (d) the total gesture instances for each gesture type.
